# Publisher Correction to: Recommendations on compiling test datasets for evaluating artificial intelligence solutions in pathology

**DOI:** 10.1038/s41379-022-01163-y

**Published:** 2022-09-23

**Authors:** André Homeyer, Christian Geißler, Lars Ole Schwen, Falk Zakrzewski, Theodore Evans, Klaus Strohmenger, Max Westphal, Roman David Bülow, Michaela Kargl, Aray Karjauv, Isidre Munné-Bertran, Carl Orge Retzlaff, Adrià Romero-López, Tomasz Sołtysiński, Markus Plass, Rita Carvalho, Peter Steinbach, Yu-Chia Lan, Nassim Bouteldja, David Haber, Mateo Rojas-Carulla, Alireza Vafaei Sadr, Matthias Kraft, Daniel Krüger, Rutger Fick, Tobias Lang, Peter Boor, Heimo Müller, Peter Hufnagl, Norman Zerbe

**Affiliations:** 1 Fraunhofer Institute for Digital Medicine MEVIS, Max-von-Laue-Straße 2, 28359 Bremen, Germany; 2 Technische Universität Berlin, DAI-Labor, Ernst-Reuter-Platz 7, 10587 Berlin, Germany; 3 Institute of Pathology, Carl Gustav Carus University Hospital Dresden (UKD), TU Dresden (TUD), Fetscherstrasse 74, 01307 Dresden, Germany; 4 Charité – Universitätsmedizin Berlin, corporate member of Freie Universität Berlin and Humboldt Universität zu Berlin, Institute of Pathology, Charitéplatz 1, 10117 Berlin, Germany; 5 Institute of Pathology, University Hospital RWTH Aachen, Pauwelsstraße 30, 52074 Aachen, Germany; 6 Medical University of Graz, Diagnostic and Research Center for Molecular BioMedicine, Diagnostic & Research Institute of Pathology, Neue Stiftingtalstrasse 6, 8010 Graz, Austria; 7 MoticEurope, S.L.U., C. Les Corts, 12 Poligono Industrial, 08349 Barcelona, Spain; 8 Lakera AI AG, Zelgstrasse 7, 8003 Zürich, Switzerland; 9 QuIP GmbH, Reinhardtstraße 1, 10117 Berlin, Germany; 10 Helmholtz-Zentrum Dresden Rossendorf, Bautzner Landstraße 400, 01328 Dresden, Germany; 11 Olympus Soft Imaging Solutions GmbH, Johann-Krane-Weg 39, 48149 Münster, Germany; 12 Tribun Health, 2 Rue du Capitaine Scott, 75015 Paris, France; 13 Mindpeak GmbH, Zirkusweg 2, 20359 Hamburg, Germany

**Keywords:** Software, Pathology

Correction to: *Modern Pathology* 10.1038/s41379-022-01147-y, published online 10 September 2022

The “Competing interests” section was erroneously not transferred from the manuscript to the originally published version of the article. The “Competing interests” section should read: F.Z. is a shareholder of asgen GmbH. P.S. is a member of the supervisory board of asgen GmbH. All other authors declare that they have no conflict of interest. The original article has been corrected accordingly.

